# Passive hallux adduction decreases lateral plantar artery blood flow: a preliminary study of the potential influence of narrow toe box shoes

**DOI:** 10.1186/s13047-019-0361-y

**Published:** 2019-11-04

**Authors:** Julia L. Jacobs, Sarah T. Ridge, Dustin A. Bruening, K. Annie Brewerton, Jayson R. Gifford, Daniel M. Hoopes, A. Wayne Johnson

**Affiliations:** 1 Department of Exercise Sciences, Brigham Young University, 106 Smith Fieldhouse, Provo, UT 84602 USA; 2 Revere Health Orthopaedics, 1055 North 500 West #121, Building C, Provo, UT 84604 USA

**Keywords:** Plantar fasciitis, Narrow footwear, Plantar fascia, Hallux valgus, Vascular ultrasound, low arch

## Abstract

**Background:**

Blood flow is essential in maintaining tissue health. Thus, compromised blood flow can prevent tissue healing. An adducted hallux, as seen inside a narrow shoe, may put passive tension on the abductor hallucis, compressing the lateral plantar artery into the calcaneus and restricting blood flow. The purposes of this study were to compare lateral plantar artery blood flow before and after passive hallux adduction and to compare blood flow with arch height.

**Methods:**

Forty-five healthy volunteers (20 female, 25 male; age = 24.8 ± 6.8 yr; height = 1.7 ± 0.1 m; weight = 73.4 ± 13.5 kg) participated in this cross-over design study. Arch height index (AHI) was calculated, and blood flow measurements were obtained using ultrasound (L8-18i transducer, GE Logiq S8). The lateral plantar artery was imaged deep to abductor hallucis for 120 s: 60 s at rest, then 60 s of passive hallux adduction. Maximal passive hallux adduction was performed by applying pressure to the medial side of the hallux. Blood flow was calculated in mL/min, and pre-passive hallux adduction was compared to blood flow during passive hallux adduction.

**Results:**

Log transformed data was used to run a paired t-test between the preadduction and postadduction blood flow. The volume of blood flow was 22.2% lower after passive hallux adduction compared to before (− 0.250 ± 0.063, *p* < 0.001). As AHI decreased, there was a greater negative change in blood flow. As baseline blood flow increased, there was also a greater negative change in blood flow.

**Conclusions:**

Our preliminary findings of decreased blood flow through passive hallux adduction indicate conditions that elicit passive hallux adduction (e.g. wearing narrow-toed shoes) may have important effects on foot blood flow. Individuals with lower AHI appear to have a greater risk of decreased blood flow with passive hallux adduction.

## Background

The posterior tibial artery supplies blood to the plantar region of the foot, including the plantar fascia (1, 2). Plantar fasciitis is a highly prevalent injury and the most common cause of plantar heel pain (3, 4). The pathology accounts for one million medical visits in the United States each year (4). However, in spite of its frequent diagnosis, plantar fasciitis remains poorly understood (4). Research suggests that the cause of plantar fasciitis may be multifactorial (5, 6). These factors may include repetitive strain on the plantar fascia, (5–8) intrinsic foot muscle weakness (9), calf tightness (5), improper shoes (5, 10), pes planus (5–8, 10), pes cavus (5, 6, 8) and excessive pronation (6, 7). It has also been suggested that plantar fasciitis may be accompanied by degenerative changes rather than inflammation (11). Given the importance of blood flow in tissue healing, blood flow may help explain the 46% recurrence rate of plantar fasciitis reported in previous research (12).

Few studies have examined blood flow to the plantar fascia with regard to healing. Blood flow is essential following injury as it provides the vital reparative factors for healing (13). For example, anatomical structures such as the scaphoid and patella have difficulty healing due to poor vascularization (14, 15). Previous research has shown that decreased blood flow can also prevent healing in the Achilles tendon (16). Due to the continuity of the plantar fascia and Achilles tendon, (17–20) it can be assumed that decreased blood flow may be preventing healing not only of the Achilles tendon but of the plantar fascia as well. In the plantar fascia, however, the lack of blood flow may be augmented by footwear.

Modern footwear, which is often tight or narrow (21), can laterally deviate the hallux (22) which may consequently affect blood flow. A laterally deviated hallux may place the posterior tibial artery at risk for partial occlusion from impingement. Prior to supplying blood to the plantar aspect of the foot, the artery passes posterior and inferior to the sustentaculum tali of the calcaneus and then divides into medial and lateral plantar arteries deep to the abductor hallucis (1). There is a possibility of impingement against the calcaneus if the abductor hallucis were to be enlarged, contracted or passively tensed as would be seen during passive hallux adduction. Eighty-eight percent of women and a large proportion of men have been shown to wear ill-fitting shoes (21, 22). Due to the narrow toe box featured in modern footwear (21), lateral deviation of the hallux may be a common phenomenon, making its effects on blood flow worth investigating.

In addition to adduction of the hallux, arterial impingement may be influenced by overall foot structure. A pes cavus foot, for example, may have tighter muscles due to the shortened medial longitudinal arch (23). A tight and shortened abductor hallucis could be more likely to occlude the posterior tibial artery that runs deep to it. Also, pes planus may put excessive stretch or tension on the abductor hallucis, which could theoretically put pressure on the posterior tibial artery (23). Previous studies on plantar fasciitis have shown that pes planus and pes cavus foot structures are associated with higher plantar fasciitis incidence rates, although this has been thought to be influenced by altered foot mechanics (5, 6, 8, 24). No studies have considered the influence that these structural deformities may have on blood flow.

Thus, the purpose of this preliminary study was to 1) examine the effects of passive hallux adduction on blood flow in healthy subjects and 2) determine if changes in blood flow are related to arch height. We theorized that lateral deviation of the hallux would contribute to a decrease in the amount of lateral plantar artery blood flow (the larger of the two branches from the posterior tibial artery) and that the decrease in blood flow would be greatest in the low-arch and high-arch individuals.

## Methods

Forty-five healthy volunteers (20 female, 25 male; age = 24.8 ± 6.8 yr; height = 1.7 ± 0.1 m; weight = 73.4 ± 13.5 kg) participated in the present study. All subjects were free of lower extremity injury or pain at the time of the study and had been for at least 3 months as determined by self-report on an IRB approved intake form. Subjects were excluded if they had moderate to severe hallux valgus. Hallux valgus deformity was defined using the grading scale established by Garrow et al. (25). Prior to participation, written informed consent was obtained from all subjects, and study procedures were approved by the university’s Institutional Review Board (Review board number: X17492) and in compliance with the principles of the Declarations of Helsinki.

Participants’ shoes were removed, and seated foot posture measurements of the right foot were obtained using the Arch Height Index Measurement System (Jaktool Engineered Solutions, Cranbury, NJ) (26, 27). Arch height index (AHI) was then calculated by dividing dorsum height by truncated foot length (26, 27).

Ultrasound imaging was performed in a temperature-controlled room. Upon arrival, subjects removed their shoes and socks to allow acclimatization to the testing room. Subjects then sat quietly and completed the consent form and other paper work and had their AHI measured (~ 20 min). Immediately, prior to ultrasound imaging, each subject lay supine on a treatment table for a further 5 min in order to allow blood flow to normalize. Each subject was positioned with a pillow under the externally rotated right leg. Electrocardiogram (ECG) electrodes were adhered to the skin over the right and left chest and lower right abdomen. Ultrasound images were then recorded of the right foot before and after the initiation of maximal passive hallux adduction. The images were taken by a licensed ultrasound technician with 4 years of vascular sonography experience. The reliability of this sonographer was found to be excellent with an Intraclass Correlation Coefficient (Two-Way mixed with random subjects and fixed scanner) score of 0.99 between repeated measurements of blood flow volume.

**Fig. 1:**
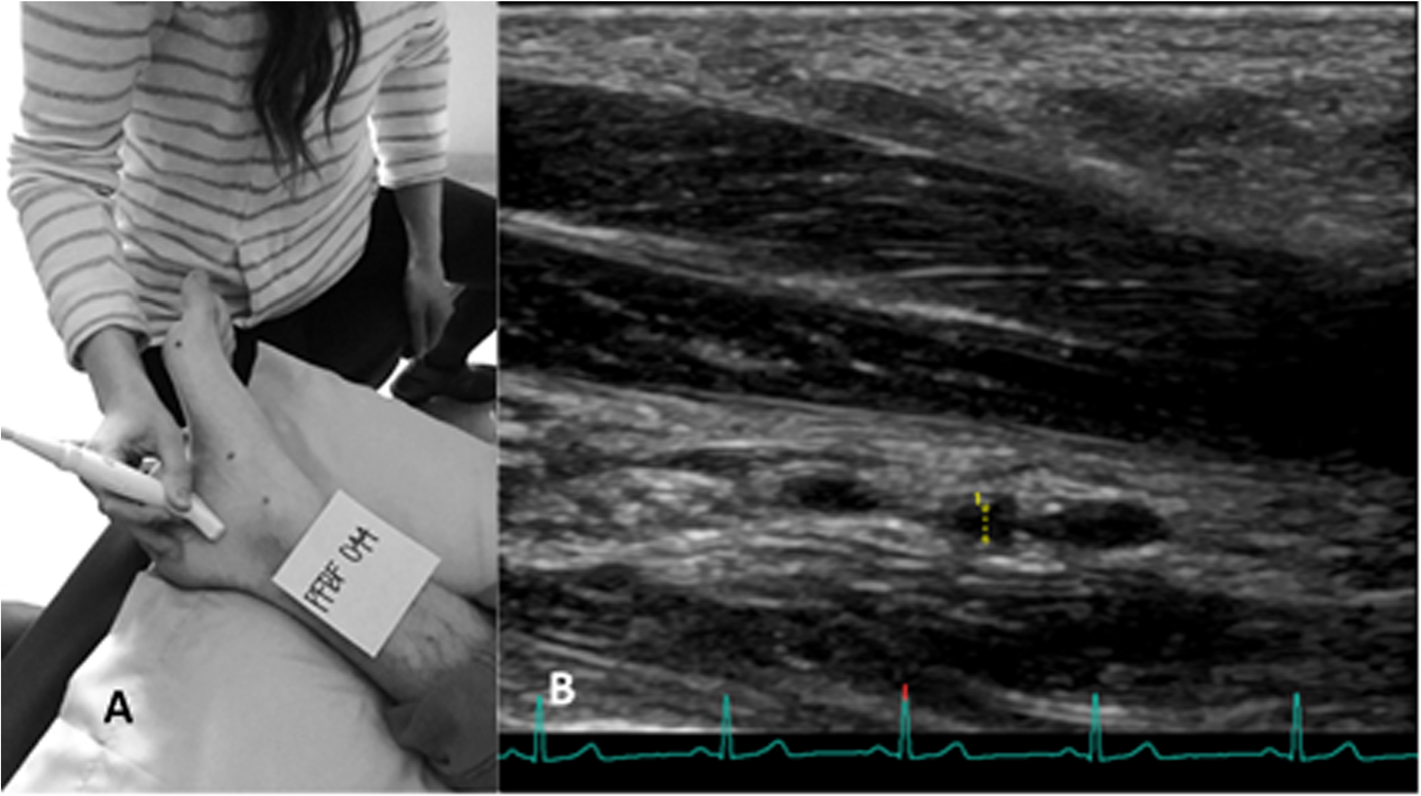
**a**) The position of the L8-18i transducer recording a longitudinal image of the lateral plantar artery. Images were taken deep to abductor hallucis. **b**) The lateral plantar artery diameter was measured at the widest part of the artery from one tunica intima to the other as indicated on this image by the dotted vertical line

Vessel diameter and blood velocity measurements were obtained from the lateral plantar artery deep to the abductor hallucis using a Logiq S8 ultrasound system (Fig. 1; General Electric Company, Fairfield, CT). Real-time ultrasound images were recorded using an L8-18i linear transducer. Transverse images were taken of the vessel in brightness mode (B-mode) before and after the initiation of passive hallux adduction. Longitudinal images were also taken of the vessel before and during passive hallux adduction, but were taken in triplex mode: color flow, pulse-wave, and B-mode.

**Fig. 2:**
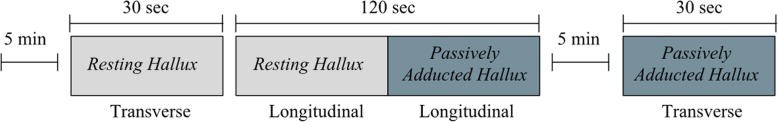
Ultrasound imaging protocol. Longitudinal images were taken in triplex mode (B-mode, color flow and pulse-wave modes) to obtain blood velocity. Transverse images were taken in B-mode to obtain vessel diameter measurements

The same imaging protocol was used for each subject (Fig. 2). Following the initial 5-min rest period, the lateral plantar artery was imaged from a transverse view at rest for 30 s. Immediately following the transverse video, the transducer was turned 90 degrees, and the lateral plantar artery was imaged longitudinally in triplex mode for 120 s. The first 60 s of the longitudinal video was taken at rest while the subsequent 60 s of the video was taken during sustained passive hallux adduction. At the completion of the 120-s video, the hallux was released, and the subject lay at rest for another 5 min. The lateral plantar artery was then imaged transversely for 30 s during sustained passive hallux adduction.

**Fig. 3:**
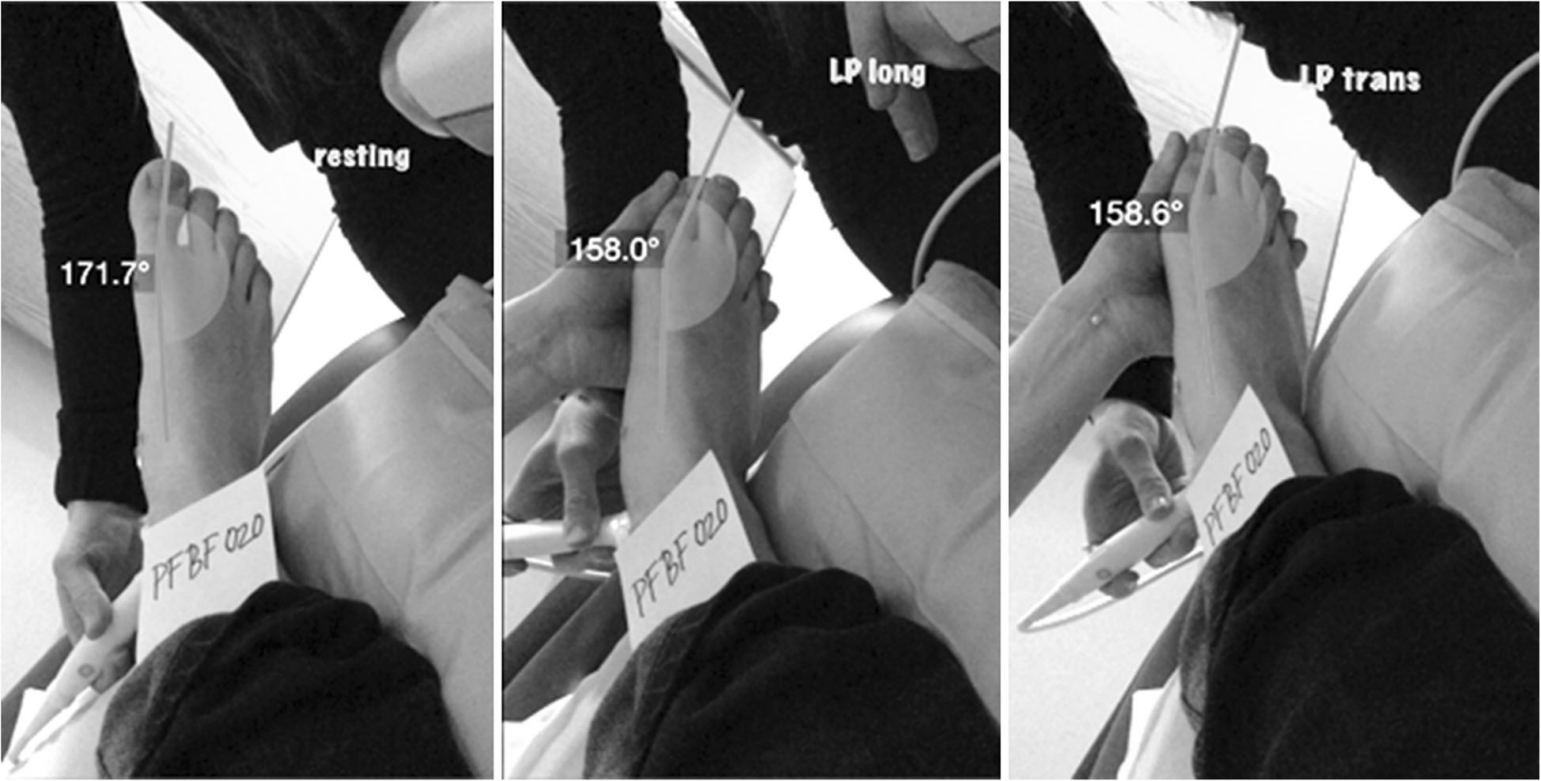
Photographs of each subject’s foot were taken at rest (left photo) and during both of the adducted hallux phases (center and right photos) of the ultrasound protocol. First metatarsophalangeal joint angles were then measured using Dartfish software

Maximal passive hallux adduction was applied to each subject by the same investigator. Passive hallux adduction was performed by applying pressure to the medial side of the proximal phalanx of the hallux enough to achieve full frontal plane range of motion at the first metatarsophalangeal (MTP) joint without causing discomfort to the subject. The lateral toes were slightly passively extended when necessary in order to allow unobstructed lateral movement of the hallux. Photographs were taken of the hallux at rest and during each passive hallux adduction condition in order to measure the amount of passive hallux adduction in each subject (Fig. 3).

Following data collection, Dartfish Video Software (Fribourg, Switzerland) was used to measure the adduction angle of the hallux before and during both passive hallux adduction trials. The angle of adduction was measured at the first MTP joint. The origin of the angle was placed over the MTP joint, and the ends were aligned with the first metatarsal proximally, and distally bisected the toenail (Fig. 3). The difference between each subject’s resting toe angle and the average of the two maximally adducted angles was then calculated to determine the amount of angle change.

Blood velocities were automatically computed from the longitudinal ultrasound videos. In pulse-wave mode, the ultrasound system uses Doppler technology to measure blood velocity. Due to the natural ebb and flow of blood within a vessel between heartbeats, the ultrasound system outputs an average blood velocity for each cardiac cycle. Average blood velocity was recorded for each cardiac cycle throughout the 120-s longitudinal video.

The B-mode, transverse videos were used to obtain the lateral plantar artery diameter. This was not done in the longitudinal videos due to the difficulty of imaging such a small vessel. We used the transverse view to ensure the true diameter was measured. The measurement calipers within the ultrasound software were used to measure the vessel diameter manually. The measurement was taken at the widest part of the artery from one tunica intima to the other (Fig. 1). The integrated ECG data was used in order to allow for consistent measurement of vessel diameter throughout each cardiac cycle. Diameter was measured at end-diastole as represented by the peak of the R wave (28). Vessel diameter was measured and recorded for each cardiac cycle throughout the 30-s resting and 30-s adducted videos.

Due to the variability between average blood velocity measurements, a 3-s rolling average was used to smooth the velocity data over the resting and passive hallux adduction conditions within each subject.^12^ Vessel diameter was averaged over the 30-s resting trial to obtain a preadduction diameter. The postadduction diameter was obtained by averaging the vessel diameters over the 30-s passive hallux adduction trial. The following equation was then used with the average blood velocity measurement for each heartbeat in order to calculate blood flow in milliliters per minute:

$$ \mathrm{Blood}\ \mathrm{Flow}={\mathrm{Velocity}}_{\mathrm{mean}}\bullet \uppi \bullet {\left(\frac{\mathrm{vessel}\ \mathrm{diameter}}{2}\right)}^2\bullet 60 $$

**Fig. 4:**
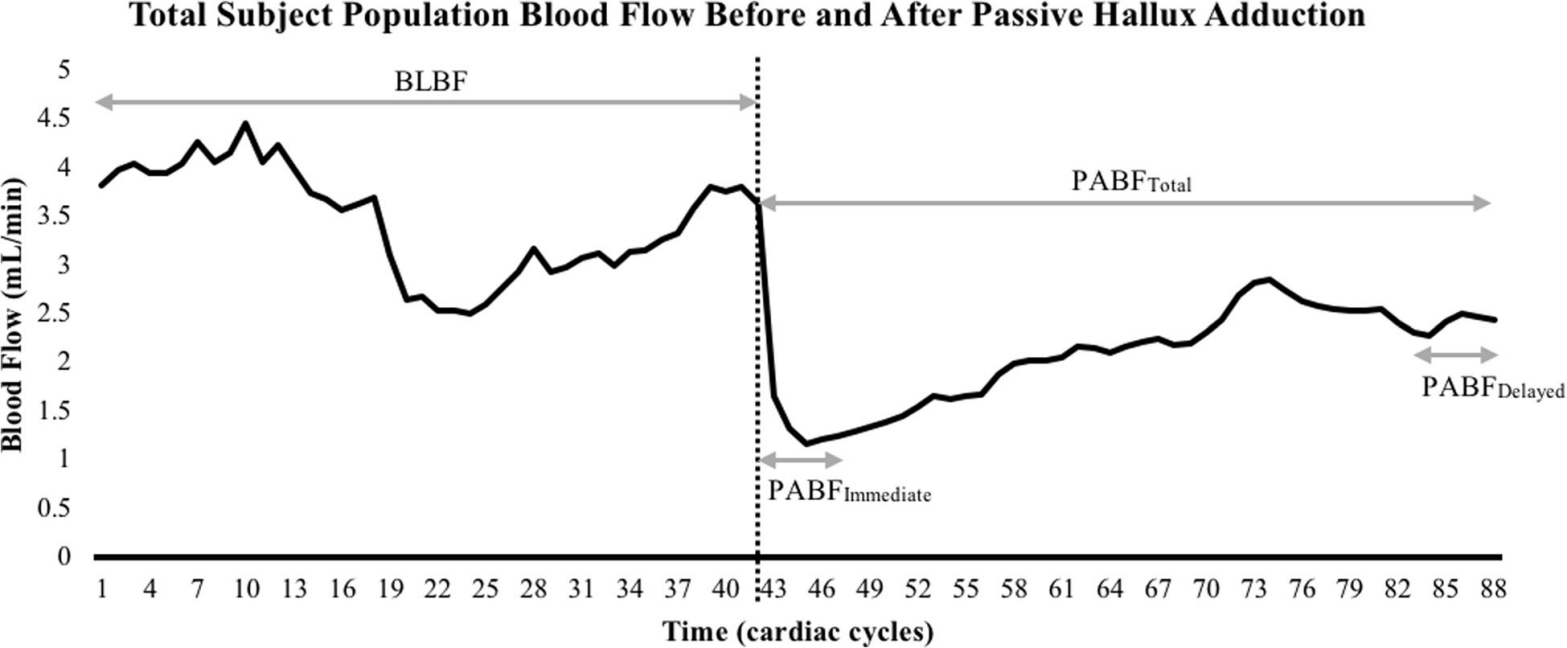
Baseline (BL) blood flow, overall postadduction (PA_Total_) blood flow, average blood flow over the 5 cardiac cycles immediately following passive hallux adduction (PA_Immediate_) blood flow, and average blood flow over the 5 cardiac cycles at the end of passive hallux adduction (PA_Delayed_) blood flow. Black dotted line crossing the center of the x-axis indicates the point of passive hallux adduction

Preadduction or baseline (BL) blood flow was calculated for each subject by averaging blood flow over the entire 60-s resting condition. Postadduction blood flow (PA_total_) was calculated by averaging blood flow over the 60-s passive hallux adduction condition. To further examine the difference between BL blood flow and PA_total_ blood flow, the blood flow was averaged over 5 cardiac cycles immediately following adduction (PA_Immediate_) blood flow and the final 5 cardiac cycles of the passive hallux adduction trial (PA_Delayed_) blood flow (Fig. 4).

Blood pressure was measured at rest and during passive hallux adduction in a pilot study using photoplethysmography. It was determined that there was no significant change in blood pressure, thus blood pressure was not measured in the present study.

To determine differences between BL blood flow and PA_Total_ blood flow, a paired t-test was used. However, physiological differences in blood flow suggested the need for a natural log transformation in order to account for the skewness in flow across subjects. A second paired t-test was then used to compare lnBL blood flow and lnPA_Total_.

In order to address the question of whether or not AHI had an influence on the change in blood flow, a log transformation was again used. The change in blood flow can be represented as the natural log of the ratio of PA_Total_ blood flow over BL blood flow. Stepwise multiple regressions (forward and backward) were then used to determine if there were any variables which significantly influenced change in blood flow. Subject height, weight, age, foot length, AHI, heart rate, resting toe angle, adducted toe angle, average toe angle change between resting and adducted positions, and BL blood flow were all used as variables in the stepwise regression analysis.

To better understand the change in blood flow, a repeated measures analysis of 3 time points was used. The analysis was run using BL blood flow, PA_Immediate_ blood flow and PA_Delayed_ blood flow. To test whether there was a difference in vessel diameter before and after passive hallux adduction or a difference in the adducted toe angle between transverse and longitudinal ultrasound trials, paired t-tests were used. Means and standard deviations were calculated for all variables. Statistical tests were run in SAS (SAS Institute, Inc., Cary, NC) with alpha set to 0.05 for significance.

## Results

Prior to using a log transformation, there was no significant difference found between BL blood flow and PA_Total_ blood flow (BL blood flow = 3.53 ± 8.93 mL/min, PA_Total_ blood flow = 2.16 ± 4.49 mL/min, *p* = 0.079). There was, however, a significant difference found between lnBL blood flow and lnPA_Total_ blood flow (− 0.250 ± 0.063, *p* < 0.001). This difference in the log transformed blood flow can be represented by a 22.2% decrease in overall blood flow before and after passive hallux adduction.

Two variables in the regression were found to have a significant influence on change in blood flow: AHI (*p* = 0.04) and BL blood flow (*p* = 0.023). As AHI decreased, there was a greater negative change in blood flow. As BL blood flow increased, there was also a greater negative change in blood flow.

When comparing additional time points to further examine the blood flow curve (Fig. 4), BL blood flow was found to be significantly different from both PA_Immediate_ blood flow (BL blood flow = 3.53 ± 8.93 mL/min, PA_Immediate_ blood flow = 1.36 ± 2.06 mL/min, *p* < 0.001) and PA_Delayed_ blood flow (PA_Delayed_ blood flow = 2.42 ± 5.48 mL/min, *p* = 0.008). An immediate 60% decrease in blood flow was found between BL blood flow and PA_Immediate_ blood flow, while a 29% decrease in blood flow was found between BL blood flow and PA_Delayed_ blood flow. A significant difference was also found between PA_Immediate_ blood flow and PA_Delayed_ blood flow (*p* = 0.014). This difference can be represented by a 31% increase in blood flow from PA_Immediate_ blood flow to PA_Delayed_ blood flow.

There was a significant difference in vessel diameter between pre-passive hallux adduction and post-passive hallux adduction (pre = 0.129 ± 0.05 cm, post = 0.120 ± 0.05 cm, *p* < 0.001). However, adducted toe angles were not significantly different between transverse and longitudinal ultrasound trials (transverse = 29.1 ± 4.7°, longitudinal = 28.6 ± 4.2°, *p* = 0.08). The means and standard deviations of participants’ age, height, weight, foot length, AHI, resting toe angle, adducted toe angle, toe angle change, and heart rate are represented in Table 1.

**Table 1:** Demographic characteristics with mean (SD)

	Overall
Age (year)	24.8 (6.8)
Height (m)	1.73 (0.10)
Weight (kg)	73.4 (13.46)
Foot Length (cm)	25.2 (1.78)
AHI	0.372 (0.34)
Low Arch *n* = 15	0.341 (0.006)
Mid-range Arch *n* = 15	0.368 (0.005)
High Arch *n* = 15	0.407 (0.004)
Resting Toe Angle	16.1° (4.8)
Adducted Toe Angle	28.7° (4.4)
Toe Angle Change	12.7° (3.7)
Heart Rate	62.8 (8.8)

## Discussion

**Fig. 5:**
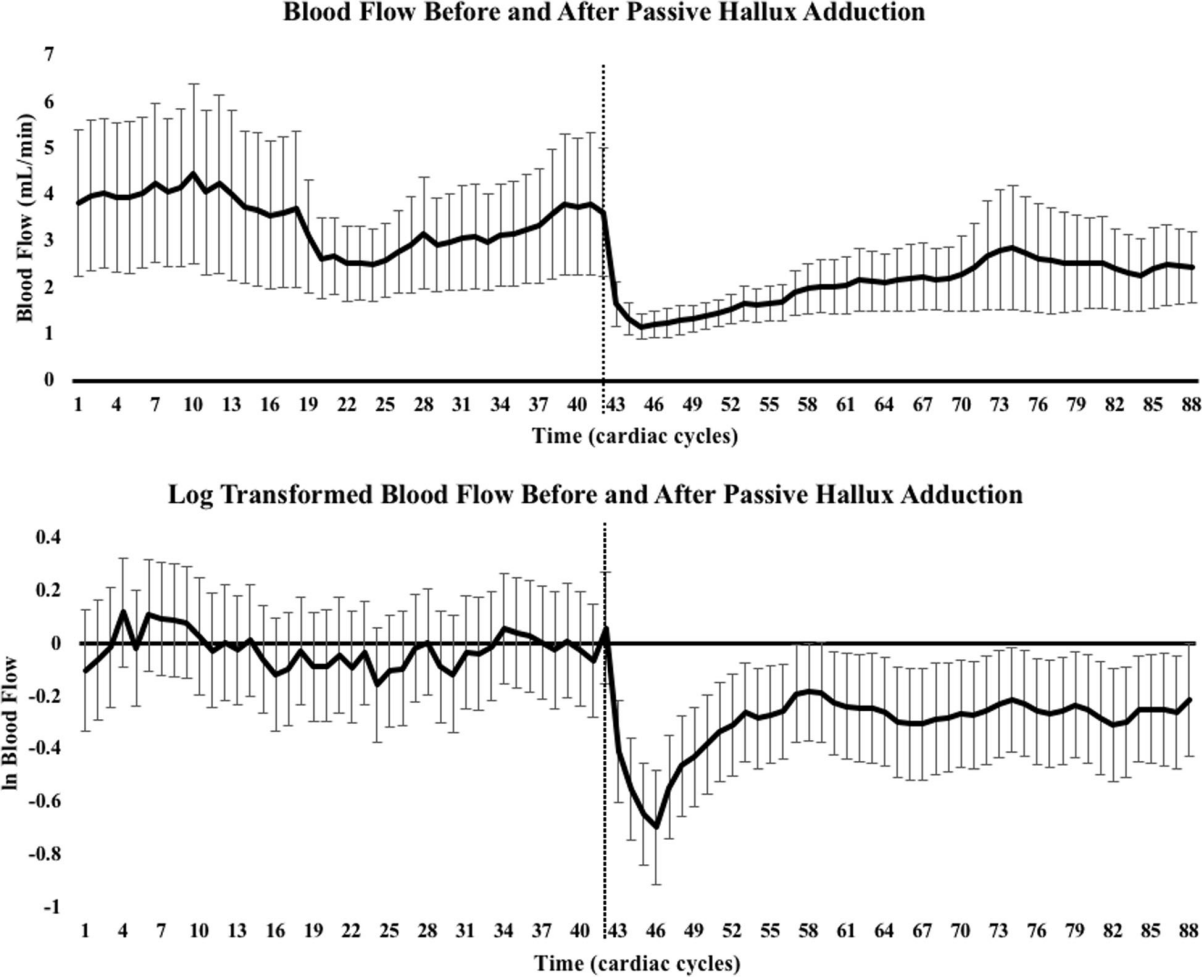
Blood flow before and after passive hallux adduction (top). Log transformed blood flow before and after passive hallux adduction (bottom). Dotted lines crossing the center of the x-axis indicate initial adduction of the hallux. Error bars show standard error

The purpose of this study was to 1) examine the effects of passive hallux adduction on blood flow and 2) determine if changes in blood flow were related to arch height. We found that due to the skewness in blood flow among our subjects, it was necessary to use a natural log transformation to account for the skewed data. After implementing the log transformation, standard error throughout the trial reduced considerably (Fig. 5), and we noted a significant decrease in overall blood flow after passive hallux adduction compared to before.

The significant decrease in both blood flow and vessel diameter shown in our results suggest that passive adduction of the hallux influenced a change in blood flow volume. This was to be expected as the posterior tibial artery, which feeds the lateral plantar artery, runs deep to the abductor hallucis (1). Passive adduction of the hallux results in an elongation or tensing of the abductor hallucis. This tension in the muscle may have compressed the artery into the calcaneus contributing to a decrease in vessel diameter, consequently decreasing blood flow.

Although passive hallux adduction was only a simulation of what would happen to the foot inside of a narrow shoe, the results of this study are suggestive that tight or narrow footwear could theoretically have an influence on blood flow within the foot. Narrow footwear is known to laterally deviate the hallux. Many shoes also simultaneously compress the foot in multiple directions, which may influence overall blood flow in the foot and reduce the potential for compensatory increased blood flow in collateral arteries. A decrease in blood flow, due to footwear or otherwise, could have a negative impact on tissue health (13) and healing as has been seen with the Achilles tendon (16), scaphoid (14) and patella (15). (12). Other conditions affected by decreased blood flow, such as pathologies (e.g. diabetes, insensate/ischaemic foot, plantar fasciitis) or healing from wounds, ulcers, and/or surgery could be further exacerbated by narrow, tight, and/or ill-fitting footwear. In plantar fasciitis, decreased blood supply to the plantar fascia could prevent full recovery and help account for the 46% recurrence rate seen in patients with plantar fasciitis. Future research should explore the effects of footwear causing passive hallux adduction on tissue health and healing.

Although the results of our study did indicate an overall decrease in blood flow to the plantar fascia, the blood flow response can be better understood after taking a closer look at the blood flow response curve (Fig. 4). Our findings indicated an overall decrease in blood flow from before to after passive hallux adduction; however, the blood flow response varied throughout the passive hallux adduction trial. The overall 22.2% decrease in blood flow (PA_Total_ blood flow) was not as drastic as the initial 60% drop in flow immediately following adduction of the hallux (PA_Immediate_ blood flow). This suggests that the body was able to adapt to the change in flow, hence the 31% increase in blood flow from PA_Immediate_ blood flow to PA_Delayed_ blood flow. However, the amount of adaptation following the immediate response varied greatly between individuals.

**Fig. 6:**
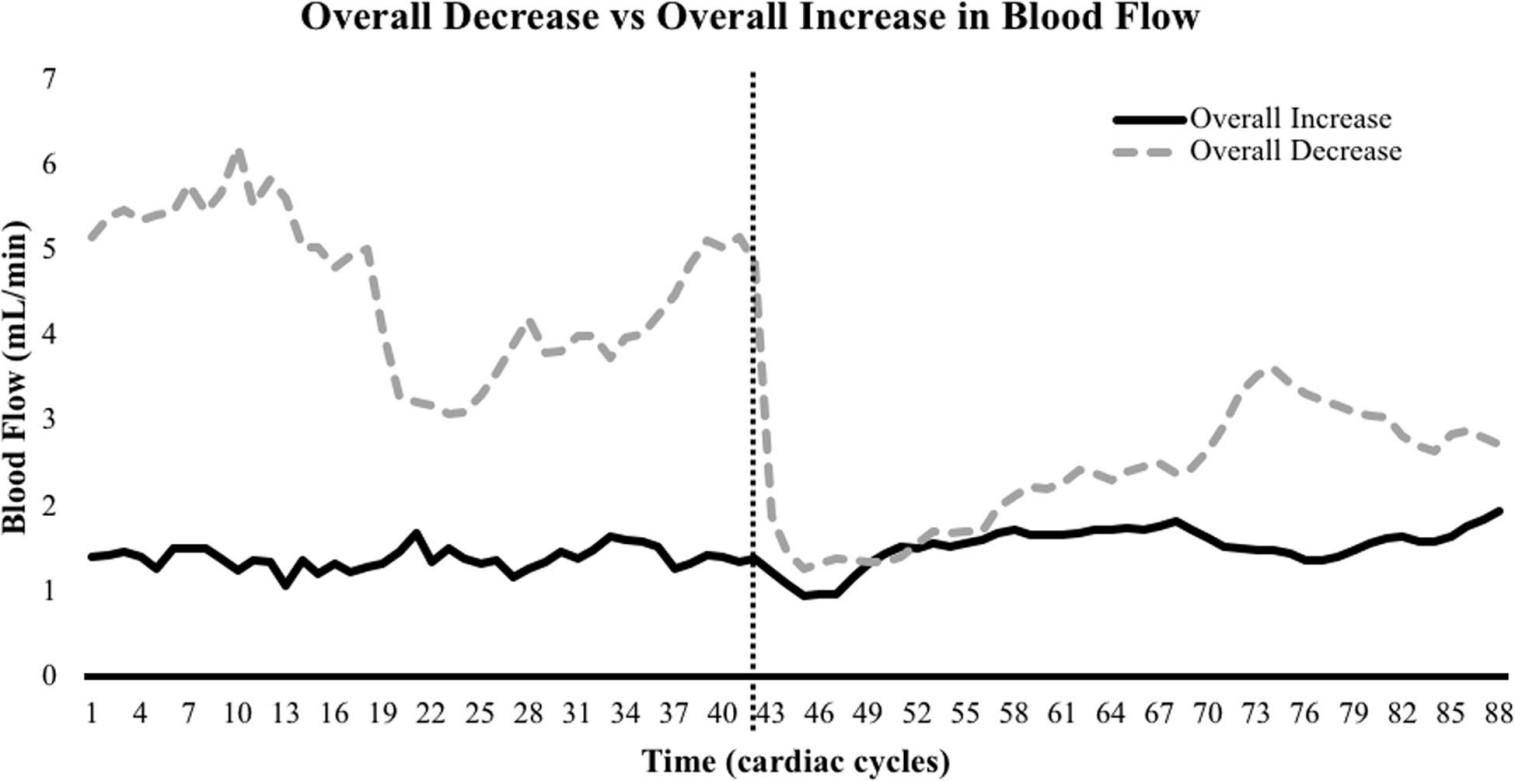
There were 29 subjects with an overall decrease in blood flow after passive hallux adduction (gray dashed line); 16 subjects had an overall increase in blood flow after passive hallux adduction (black solid line). Blood flow is represented by the 42 cardiac cycles before passive hallux adduction and 46 cardiac cycles during passive hallux adduction. Dotted black line crossing the center of the x-axis indicates the start of passive hallux adduction

Although the overall blood flow across subjects significantly decreased after passive hallux adduction, approximately one-third of the individuals actually had an overall increase in blood flow. Upon further examination of these individuals’ blood flow curves, it was determined that they collectively still had an initial decrease in blood flow but appeared to overcompensate by the end of the passive hallux adduction trial, resulting in an overall increase in blood flow (Fig. 6). This suggests that some individuals’ posterior tibial arteries responded to and were ultimately affected by passive hallux adduction while others compensated quickly, likely through vasodilation of the artery. For those whose vessels compensate quickly, passive hallux adduction may not have a noticeable effect on blood flow. These findings suggest the two-thirds of the subject population which decreased in flow without returning to normal blood flow are perhaps at a higher risk of suffering from a lack of blood flow and consequently tissue healing. Among these, several individuals showed little to no recovery in blood flow, which may represent individuals most likely to develop pathologies or have higher recurrence rates. Our results are fairly consistent with other research that showed about half of people can compensate for a reduction in blood flow, while half cannot (29). This is likely due to neurological status or variations in arterial stiffness. Future research should aim to investigate the effects of prolonged passive hallux adduction on blood flow to the plantar fascia in order to determine whether or not decreased blood flow will remain decreased.

Several factors contribute to the development of plantar fasciitis, two of which are pes planus and pes cavus feet (5, 6). Although pes planus and pes cavus have typically been described to have a mechanical influence on the development of plantar fasciitis (6), we theorized that foot structure may also affect the pathology through blood flow. Thus, we hypothesized a greater decrease in blood flow would be seen in both the high-arch individuals and low-arch individuals. Our results, however, indicated a greater decrease in blood flow in only the low-arch feet. This suggests that the abductor hallucis may already be lengthened to accommodate the flatter foot. Further lengthening of the abductor hallucis through passive hallux adduction would tense the muscle even more, potentially contributing to compression and partial occlusion of the posterior tibial artery. With so many other factors contributing to the development of plantar fasciitis (5, 6), it seems likely that a high-arch foot may be more susceptible to the injury for reasons other than restricted blood flow.

An additional finding was the significant influence of BL blood flow on the blood flow change. Normal blood flow varies between individuals. Our results indicated the greater BL blood flow, the greater the decrease in blood flow following passive hallux adduction. This suggests that the more blood there is flowing through a vessel, the greater the potential blood flow has to drop. If blood flow is already low, then the magnitude of the change in flow is lessened.

As can be expected with any research project, there were a few limitations in our study. We obtained average blood velocity readings and measured vessel diameter from two separate passive hallux adduction trials. We assumed that the blood flow response to passive hallux adduction would be the same between the two trials. Even though we cannot guarantee that there was an identical response, we do feel this was the most accurate way to measure the diameter of this relatively small artery. Also, due to the study design, the sonographer could not be blinded to the hallux position during data collection, but was blinded to AHI. Study participants were asked not to exercise immediately prior to data collection and they were questioned about their exercise for the day of data collection. Thirty-five of the subjects did not exercise the day of the study, while 10 did exercise with at least 5 h rest before participating in the study. We did not limit or monitor study participants’ use of caffeine. Finally, there was a substantial amount of variation in BL blood flow potentially due to the small vessel size. Variation between subjects was accounted for using the natural log transformation, but BL blood flow varied within individuals as well. We feel as though this variation was accounted for by averaging blood flow over the entire 60-s preadduction trial in order to obtain one baseline reading.

## Conclusions

In conclusion, passive hallux adduction has been shown to have a negative effect on blood flow within the foot. This decrease in blood flow was greatest in the low-arch individuals. Our preliminary findings of decreased blood flow through passive hallux adduction suggest blood flow in narrow footwear and its effects on the plantar fascia are worth investigating.

## Data Availability

The datasets used and/or analysed during the current study are available from the corresponding author on reasonable request.

## Abbreviations

AHI: Arch height index
BL: Preadduction or baseline
ECG: Electrocardiogram
MTP: Metatarsophalangeal
PA_Delayed_: Postadduction blood flow of the final 5 cardiac cycles of the passive hallux adduction trial
PA_Immediate_: Postadduction blood flow was averaged over 5 cardiac cycles immediately following adduction
PA_Total_: Postadduction total blood flow
